# Open reduction and pinning for the treatment of Gartland extension type III supracondylar humeral fractures in children

**DOI:** 10.1007/s11751-014-0198-7

**Published:** 2014-08-02

**Authors:** Ahmet Aslan, Mehmet Nuri Konya, Aykut Özdemir, Hüseyin Yorgancigil, Gökhan Maralcan, Emin Uysal

**Affiliations:** 1 Department of Orthopedics and Traumatology, Afyonkarahisar State Hospital, Orhangazi Mh. Nedim Helvacıoğlu Cd. Uydukent, 03100 Afyonkarahisar, Turkey; 2 Department of Orthopedics and Traumatology, Faculty of Medicine, Süleyman Demirel University, Isparta, Turkey; 3 Department of Orthopedics and Traumatology, Faculty of Medicine, Afyon Kocatepe University, Afyonkarahisar, Turkey; 4 Department of Emergency, Bağcılar Education and Research Hospital, Istanbul, Turkey

**Keywords:** Children, Humerus, Supracondylar fractures, Surgical approaches, Treatment results

## Abstract

In this study, we aim to evaluate the clinical and radiological results of children who were treated with four different surgical approaches. In our clinics between February 2004 and November 2012, the children who underwent surgical treatment for supracondylar humeral fractures and whose data were available with regular follow-up of at least 1 year were included in the study. Clinical outcomes were evaluated for 54 patients with Gartland type 3 extension supracondylar fractures. Functional and cosmetic results of the patients were determined according to the Flynn criteria. Mean age of the patients was 4.9 (between 2 and 14) among which 26 of them were girls and 28 were boys. Mean operation time was 45 (35–85) min. Average length of hospital stay (LHS) was 2.9 (1–7) days. Average duration of splints was 3.5 (2–6) weeks, while the average removal period of the wires was 4.6 (3–8) weeks. Mean consolidation time was 4.6 weeks (3–8). Mean follow-up was 14.36 months. In our study, we performed 54 patients functional and cosmetic results. While 48 of the patients had satisfying results (excellent, good, or fair), six of them had unsatisfactory (poor) results. The results of this study suggest that clinical results with surgical treatment of Gartland type 3 extension fractures were satisfactory. However, the delay in the surgical treatment may cause a number of complications.

## Introduction

Supracondylar humerus fractures are the second common type of pediatric fractures. Supracondylar fractures are 50–60 % of all pediatric elbow fractures. In total, 85 % of these fractures are seen in children between ages of 4–11. Generally, conservative treatment options are preferred in pediatric fractures [1]. Surgical procedures are the treatment of choice in displaced supracondylar humerus fractures [2]. Humerus fractures are a significant part of pediatric fractures due to high incidence, high morbidity, and serious complications [3, 4].

Four different surgical approaches have been described in displaced supracondylar humerus fractures requiring surgical treatment [5, 6]. In the literature, every approach has its own positive aspects and there are some publications reporting good results [6–8]. Although there are comparative studies for some of these surgical approaches, we did not find any study comparing four different approaches. In this study, we aim to evaluate the clinical and radiological results of children who were treated with four different surgical approaches.

## Patients and methods

In our clinics between February 2004 and November 2012, the children who underwent surgical treatment for supracondylar humeral fracture with available data and regular follow-up of at least 1 year were included in the study. Fractures treated with closed reduction and percutaneous fixation excluded. Initial medical story and neurovascular physical examination were recorded in the emergency room for all patients. Anterior–posterior and lateral radiographs of the elbow were obtained. All the results were recorded. In some patients due to excessive displacement and poor position, we tried to ensure a closed reduction with gentle manipulation until the surgery. All of the patients were hospitalized, and long arm splints were applied. Radiographic control again was followed by the implementation of a long arm splint elbow in 90° flexion. Then, the patients were operated as soon as possible. Open reduction–internal fixation (ORIF) indications were, fractures with high risk of neurovascular injury and engagement of the distal aspect of the proximal fragment in brachial muscles and unsatisfactory closed reductions. In this study, our groups comprise only the patients who need open reduction after failed closed reduction attempts and we only analyzed open reduction and internal fixation patients who underwent closed reduction were excluded from the study. The patients underwent surgical intervention under general anesthesia, often using pneumatic tourniquet, with four different surgical approaches. Our incision choice can be changed about fracture pattern. Nerve injury, vascular injury, fracture pattern displacement, and open fractures are the major patterns of incision choice. The fractures were fixed with at least two lateral or cross Kirschner wires (K-wires) under fluoroscopy control due to fracture pattern and stability and surgeon’s preference.

### Surgical technique [5, 9–11]

#### Anterior approach

Transverse or longitudinal incision was made over the antecubital fossa. Subcutaneous tissues were dissected bluntly. With transverse incision distal, fragment’s displacement direction can be seen easily. Brachial artery was explored. If any suspicion of neurovascular injury, this is the best approach. In displaced fractures, usually the brachialis muscle is torn and the fracture can be explored easily. Soft tissue interposition was removed. The distal fragment was pulled along the proximal fragment, and the reduction was achieved by applying pressure.

#### Lateral approach

Incision was made beginning from 5 to 6 cm proximal to 2–3 cm distal to the elbow joint. Dissection was made through biceps and brachialis muscles. If there is any interposition of soft tissues, a manipulation may be required to achieve reduction.

#### Medial approach

Incision begins 5 cm above the elbow joint, medial to intermuscular septum, and just below the medial epicondyle. Nervus ulnaris was dissected and protected. Fracture line can be found by beneath the triceps and brachialis muscles. Continuity of fracture line was palpated, and reduction was achieved.

#### Posterior approach

Skin incision was made midline to olecranon starting about 5 cm proximal to the olecranon, giving a slight curve to the distal for 1–2 cm. Ulnar nerve was located to prevent an injury.

Fixation was made by at least two cross or lateral K-wires in all approach. All patients were treated according to the same postoperative protocol. A long-arm cast was applied in the elbow 90° flexion and neutral forearm rotation. Antibiotic prophylaxis with Cefazolin sodium was given 50 mg/kg, four times a day for 24 h. The sutures were removed after 10 days. Postoperative radiological controls were performed on the first, seventh, and thirtieth days. Although it is preferred to remove the K-wires until the end of 4th week, we generally removed the wires between 4th and 5th weeks. Our patients were generally coming from rural and distant areas to authors’ hospitals. Usually, patient and family compliance and cooperation were moderate or poor. To prevent some postoperative complications such as losing reduction or refracture, authors have followed some more conservative approach. Active exercises were started according to the fracture healing in radiographs. Modified criteria developed by Flynn [3] were used for evaluation (Table 1).

**Table 1:** Modified Flynn Criteria

Outcome	Rating	Cosmetic factor (carrying angle loss in degrees)	Functional factor (movements loss in degree)
Satisfactory	Excellent	0–5	0–5
	Good	6–10	6–10
	Fair	11–15	11–15
Unsatisfactory	Poor	>15	>15

### Statistical analysis

Statistical analysis was performed using SPSS statistical package program (SPSS 19.0 version, SPSS Inc., Chicago, Illinois, USA). Kolmogorov–Smirnov test was used for normal distribution of the data. Pearson’s chi-square tests were used in significance analysis. Also we have done power analysis for Pearson’s Chi-square test.

## Results

In total, 28 patients (52 %) were male and 26 (48 %) were female. The mean age was 4.9 years. The patients were distributed between the ages of two and 14. The peak range was between 4 and 8 years of age (58.7 %). The fractures were at the right elbow in 54 % of the cases and left elbow in 46 % of the cases. The most common admissions were in the spring season with 22 cases (40.0 %), mostly in May with 19 patients (30 %). Falls (in-house, out of house, and falls from height) were the most common injuries (96 %). All children first get a trial of closed reduction and pinning if the reduction is adequate. Only nine children were immediately taken for open reduction based on presentation nerve injury, vascular injury, fracture pattern displacement, excessive swelling, and previous bonesetter’s bad intervened.

The majority of patients (75 %) were operated in the first 24 h. In 25 % of the cases, the time between injury and surgical intervention was more than 24 h, for various reasons. Five of all patients had accompanying injuries. Two had ipsilateral fractures of the distal radius, and the others had a first metacarpal basis fracture, a contralateral forearm both bone fractures, and a tibial spiral-oblique fracture. Four different incisions were preferred. Circulatory status of the skin, condition of the fracture fragments, and surgeons’ preference has been effective in choice of the incision. Mean operation time was 45 (35–85) min. Average LHS was 2.9 (1–7) days. Average duration of splints was 3.5 (2–6) weeks, while the average removal period of the wires was 4.6 (3–8) weeks. Mean consolidation time was 4.6 weeks (3–8). Mean follow-up was 14.36 months. We have made radiological assessment including an AP and lateral X-ray of elbow for all of our patients at postoperative consolidation time and at the final follow-up. The Baumann angle was measured on AP radiographic view. Diaphysis-condylar angle was measured on lateral view. Mean HEW value was −0.43° at the average consolidation time and −1.23° at the last follow-up. The mean Baumann angle value was 71.9° (64°–82°) at the average consolidation time and 74.6° (64°–88°) at the last follow-up. The mean diaphysis-condylar angle was 42.3° at the average consolidation time 44.40 at the last follow-up. In clinical findings for the average loss of mobility, loss of flexion was 1.6° and loss of extension was 0.8°.

Many scoring systems have been used for elbow disorders [12]. Our functional and cosmetic results performed by Flynn’s Criteria. Flynn criteria are obtained measuring with goniometers the range of elbow movement and the carrying angle. Carrying angle difference among both elbows angle and loss in elbow motion is scored as follows: between 0 and 5^°^, excellent; 6–10^°^, good; 11–15^°^, fair; <15^°^, and poor. In our study, we performed 54 patients functional and cosmetic results. While 48 of the patients had satisfying results (excellent, good, or fair), six of them had unsatisfactory (poor) results.

In this study, we have detected power analysis follows: A sample size of 54 achieves 6 % power to detect an effect size (W) of 0.0677 using a 3° of freedom chi-square test with a significance level (alpha) of 0.05000.

### Complications

**Fig. 1:**
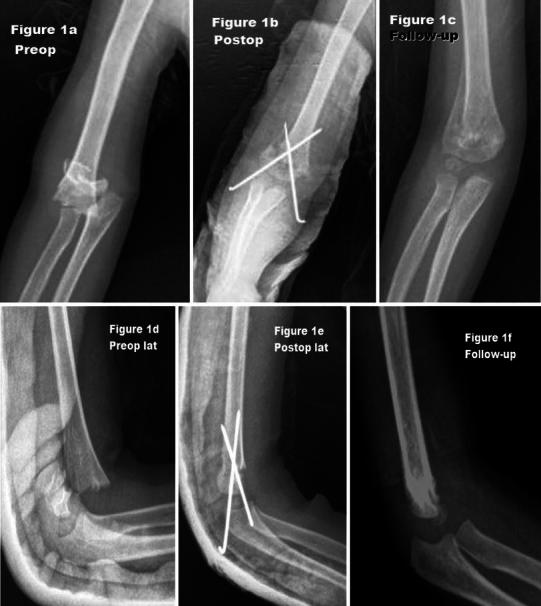
Anteroposterior (**a**–**c**) and lateral (**d**-**f**) view in preop., postop., and follow-up. Four-year-old boy, anterior approach, poor result

**Fig. 2:**
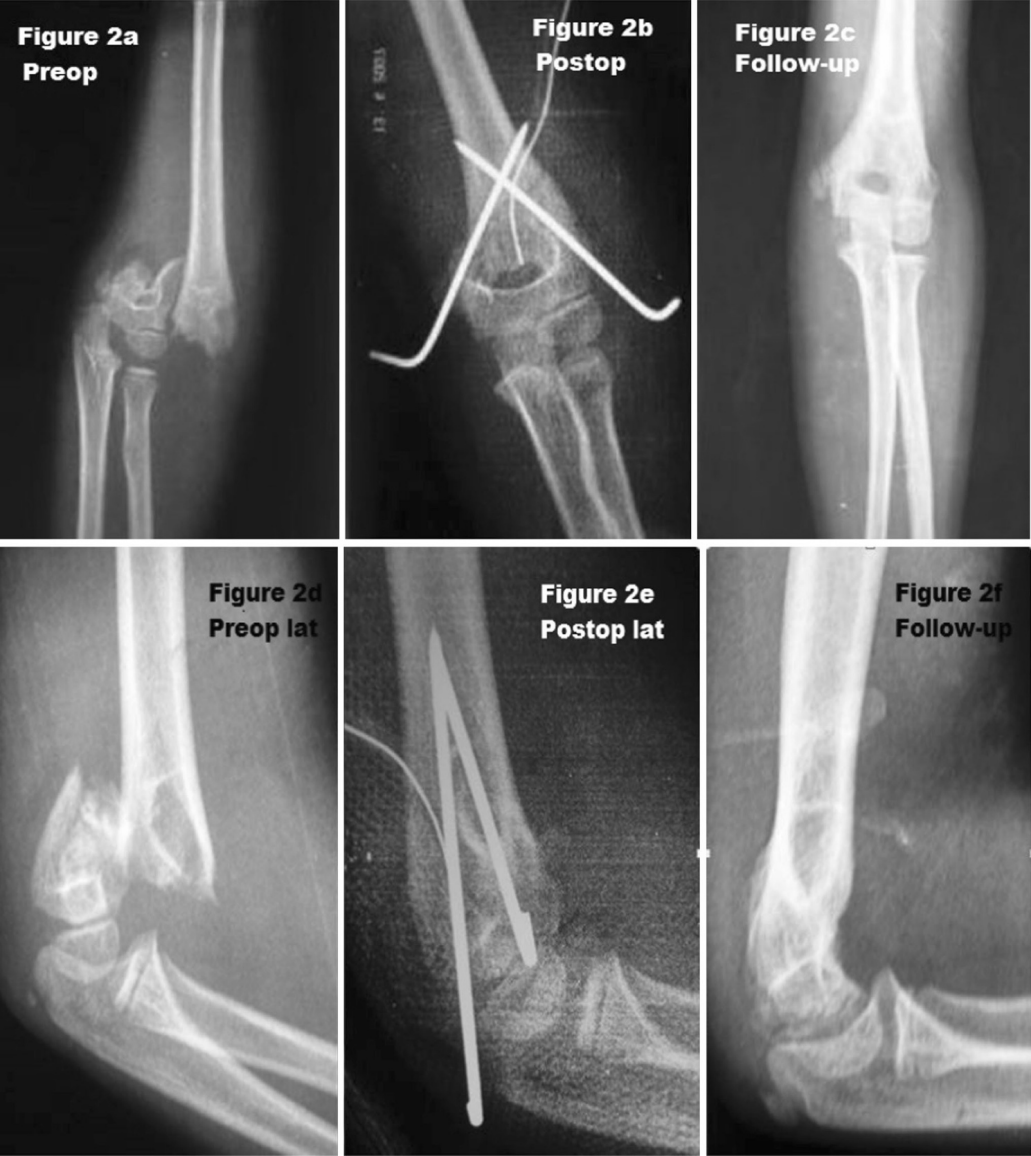
Anteroposterior (**a**–**c**) and lateral (**d**–**f**) view in preop., postop., and follow-up. Twelve-year-old boy, lateral approach, fairy result

**Fig. 3:**
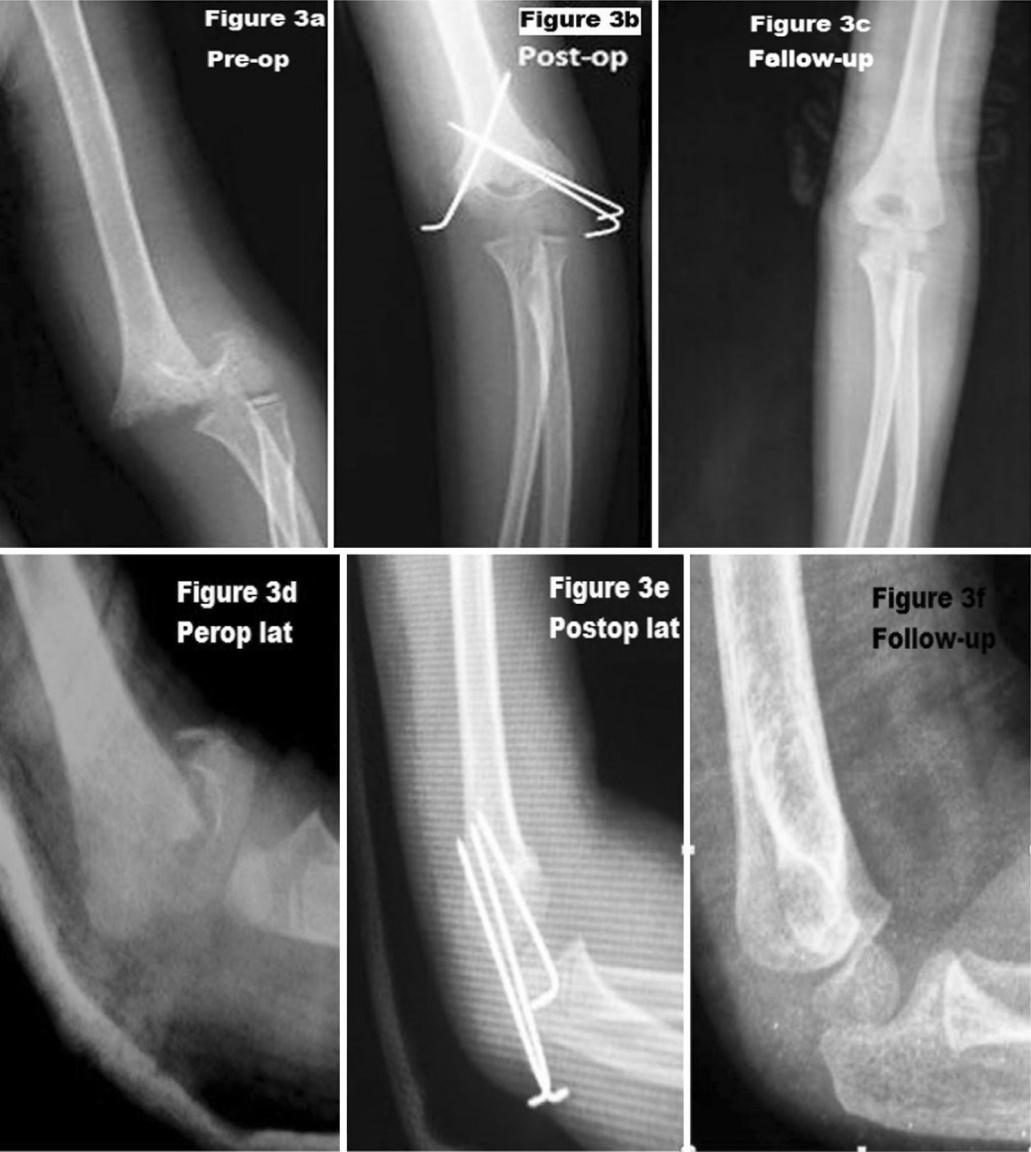
Anteroposterior (**a**–**c**) and lateral (**d**–**f**) view in preop., postop., and follow-up. Five-year-old girl, medial approach, excellent result

**Fig. 4:**
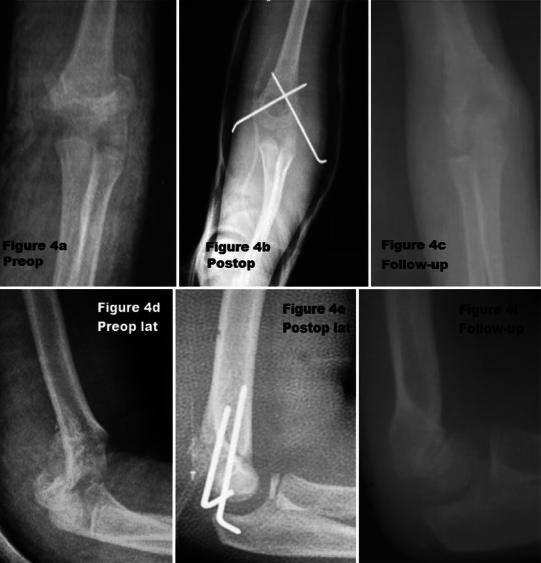
Anteroposterior (**a**–**c**) and lateral (**d**–**f**) view in preop., postop., and follow-up. Nine-year-old boy, posterior approach, good result

Preoperative and postoperative complications were observed in seven patients. Complications were more frequent in patients with longer delay than 24 h between injury and surgical intervention. This was statistically significant (*p* = 0.06). Three (5.6 %), peripheral nerve lesions were seen in the first physical examinations at admission. Four superficial pin infections (7.4 %) were found at follow-up. These were treated with oral antibiotics and appropriate dressing. At the last controls, five (9.3 %) cubitus varus deformities were noted. The patient intervened by the bonesetter was one of these patients. Some examples of our patients have shown that they have been treated with different approaches and their various results are provided in Figs 1, 2, 3, and 4. The figures including anteroposterior (a–c) and lateral (e–f) view in preop., postop., and follow-up.

## Discussion

Goals in the treatment of pediatric supracondylar humerus fractures are full recovery of elbow movements, achieving normal cosmetic view of elbow, protecting the patient from neurovascular complications that may occur. Supracondylar fractures of the humerus in children are more common under the age of 10. In particular, incidence peaks between the ages of 5–7 have been reported [4–6]. In our series, the age distribution is from 2 to 14. It has a peak incidence between 4 and 8 years of age (58.7 %), and the average age is 4.9. The mean age and the age range of peak incidence are consistent with the current literature. Supracondylar humerus fractures of childhood are more common in boys [4, 5, 13]. Archibeck et al. [14] have reported a rate of 57 % girls and 43 % boys in his series, Gosens and Bongers [15] have given the rate of 51 % female to 49 % male. In this study, 52 % were boys and 48 % were girls. Our data are consistent with the recent literature. These fractures are more frequent in boys. Boys are more active, and the games they play have a higher probability of injury.

Left elbow fractures were more common in previous studies [4, 13, 17]. Left arm handles a protective duty during a fall. In our study, right arm (54 %) was more commonly injured. There are studies with follow-up times up to 4.6 and 8.9 years [16–18]. Our study has a follow-up mean time of 14.36 months, and it may be considered adequate for screening possible complications. These fractures may be associated with other fractures. Mazda et al. [13] reported seven (6 %) ipsilateral forearm fractures in their study of 116 patients. Gordon et al. [19] reported four ipsilateral forearms, one radial neck, one distal radius, one proximal end of the humerus fracture in their series of 138 cases. Pirone et al. [16] reported 20 (8.6 %) ipsilateral forearm fractures in their series. Our study included five patients with associated fractures. Two had ipsilateral fractures of the distal radius, and the others had first metacarpal basis fracture, contralateral forearm both bone fractures, and tibia spiral-oblique fracture. Additional trauma patients were treated in the same session. Mesherle et al. [17] reported the LHS as 1.6 days in their series of 36 patients. Mulhall et al. [18] reported LSH 2.5 days in their ORIF series. Karapinar et al. [20] reported a 3.01-day LSH for 236 cases. We had a mean LSH of 2.9 days. Extension-type fractures are more common in the literature [21, 22]. Pirone et al. [16] reported a rate of 38 % type 2 and 62 % type 3 fractures. Archibeck et al. [14] reported a rate of 22 % type 1, 16 % type 2, and 61 % type 3 fractures. Our results were similar with current studies, and all of our patients had extension Gartland type 3 fractures. Supracondylar humerus fractures in children are frequently associated with various complications such as neurovascular deficit and compartment syndrome. In total, 7–16.1 % neurological injuries are reported in the literature [3, 23, 24]. Anterior interosseous nerve injuries are the most common type of nerve injuries in extension fractures, and iatrogenic ulnar nerve injury is the most common type of nerve injury in flexion-type injuries [25]. These are commonly neuropraxia-type injuries in children and generally have a good prognosis. In particular,, type 3 fractures with late admission or excessive edema on the fracture increase the possibility of iatrogenic injury during manipulation and fixation. Nerve recovery is expected in 2–6-week period up to 3 months. Iatrogenic injuries are reported to improve in the first 6 months [5, 25, 26]. One median nerve and two radial nerve involvement were noted in our study. All these patients with neurological deficits were operated after 24 h. A bonesetter had intervened in one of these cases before hospital admission. Bonesetters intervene patients frequently in our society, and major sequels may occur in patients [27]. The patient intervened by a bonesetter was followed for one week due to edema and nerve injury. Only one case was intervened by a bonesetter in our study group. In the follow-up of this patient, 10° flexion loss deformity was observed. Also, no Volkman ischemic contractures or compartment syndromes were observed.

Closed reduction and percutaneous pinning have been accepted as the gold standard in reaching these goals by many authors [28]. If close reduction cannot be achieved, open reduction should be preferred in serious displaced fractures, flexion-type fractures, nerve injury after closed reduction, open fractures requiring irrigation and debridement, in posterolateral displaced fractures with a high risk of neurovascular injury [17, 29, 30]. On the other hand, Kazimoglu et al. [31] compared primarily open reduction and internal fixation versus closed reduction and percutaneous cross-pinning of Gartland type 3 extension supracondylar fractures in children. The study performed at two different centers was 80 cases included. They reported that according to Flynn’s criteria, the outcomes of the open and closed reduction groups were not statistically significant. In conclusion, they say that closed reduction showed no superiority over open reduction. Kurer and Regan [32] evaluated open reduction of 259 cases reported by eight authors and revealed 63 % excellent, 21 % good, and 16 % poor results. In our study, the patients were treated only with surgery. While 48 of the patients had functionally satisfying results, six of them had bad results. And similarly, while 49 patients were satisfactory cosmetically, there were five poor results. In the literature, each method suggests better results than the others. We believe that the medial approach prevents iatrogenic ulnar nerve injuries, it gives a good vision ensuring the restoration of the medial column, and it is a method of the least incisional scar. The lateral approach is more secure because it is away from the neurovascular structures. The anterior approach is better in the assessment of the joint and neurovascular structures. The posterior approach is better than other approaches in manipulation of fracture fragments.

 In management of these fractures, different pin configurations were also used, adding more heterogeneity to various studies in the literature [5–8]. Yousri et al. [4] as reported in the current systematic review article: There was no significant difference between crossed and lateral pinning in terms of loss reduction. Both configurations have similar stability. Also, the authors say that there is currently no level 1 evidence comparing the outcome of crossed pinning versus lateral entry pinning in extension-type Gartland III supracondylar fracture. Mostly, we used crossed k-wires for fixation. But sometimes when it became risky for the ulnar nerve injury because of severe swelling and difficulty in pinning upon surgeon preference, two lateral pins were used.

Gennari et al. [33] reported that although the anterior approach is more technically demanding, it gives better functional results. A previous study showed that with lateral incision, postoperative range of motion was better than posterior incision. Ersan et al. [28] reported that a total of 46 patients were operated through anterior and 38 through lateral approach. According to Flynn’s criteria [3], results were excellent in 19, good in 18, and fair in one in the lateral incision group, whereas in the anterior incision group, excellent results were obtained in 31 patients and good results in 15 of them. The authors say that anterior incision when open reduction is needed in pediatric supracondylar fractures offer the advantage of a smaller scar and easy access to structures that might be injured between the fractured fragments. In the study of Eren et al. [10], a total of 40 patients with type 3 supracondylar humeral fractures were divided equally into two groups as lateral or medial approach. They reported that in the lateral approach group, functional results were excellent in 18 patients (90 %), good in one patient (5 %), and fair in one patient, while cosmetic results were excellent in 19 patients (95 %) and good in one patient. In the medial approach group, 19 patients (95 %) had excellent and one patient (5 %) had good functional results, while all the patients had an excellent cosmetic result. Authors did not find significant differences between the groups. In a study comparing different approaches, Pretell Mazzini et al. [11] reported that a combined anteromedial approach could be the method which allows the achievement of better functional and cosmetic outcome according to Flynn’s criteria. Whereas, in our study, while 48 of the patients have satisfying results, six of them have bad results at final follow-up functional assessment. There was no statistically significant difference between the four groups according to in terms of surgical approaches. And also cosmetic evaluation, while satisfactory of the 49 patients, the poor results were five and there was no statistically significant difference between the groups. In generally, K-wires can be removed 3–4 weeks after surgery in children under 10 years and in older children, it should be removed for 4–5 weeks [34]. Mean removal time of wires was 4.8 (3–8) weeks in our study. Although it is preferred to remove the K-wires until the end of 4th week, we generally removed the wires between 4th and 5th weeks. Our patients were generally coming from rural and distant areas to authors’ hospitals. Usually, patient and family compliance and cooperation were moderate or poor. To prevent some postoperative complications such as losing reduction or refracture, authors have followed some more conservative approach.

 Baumann angle is an important angle in control of the reduction. Normal range is between 64 and 81° [23]. In our study group, the mean Baumann angle was 74.6^°^. Body-condylar angle measured after the surgery shows flexion or extension displacement of the distal fracture fragment. This angle changes during skeletal maturation. Body-condylar angle changes are related with extension degrees of the elbow [35]. Normal range is 40–45^°^. In our study, we found this angle 44.4^°^. The most common complication of pediatric supracondylar fractures is cubitus varus (4–58 %). D’Ambrosia [36] revealed that cubitus varus is very rare after an adequate reduction and is related with medial angulation of the distal fragment. Ippolito et al. [22] state that varus deformity is due to the defect of the distal humeral epiphysis growth plate. Surgical intervention decreases the rate of varus deformity. Gosens and Bongers [15] reported a cubitus varus rate of 2.5 %. There were five cubitus varus cases in our study group. Cubitus valgus is not common, but associated with loss of extension and late ulnar nerve paralysis. Previous studies show a rate of 2.3 % about this complication [37]. In our study group, there was no cubitus valgus deformity. Early and delayed surgical intervention is controversial in supracondylar fractures [38]. Although the results of delayed surgical intervention are satisfactory in previous studies [39], complication rates were higher in our study group.

### Limitations

Our study was retrospective, and the groups were not equal. All operations were performed by the authors randomly. Posterior and lateral approach patients were more than the others in our series. This was due to the fact that these two approaches are more popular. We also started using the anterior approach relatively more recently. We did not assess the results with respect to the implementation of two cross or lateral K-wires. Also performance of the operations by different surgeons may have influenced the results. We think that open reduction makes the pin placement some more difficult because of a desire to work within or around incisions. As can be seen in our cases pictures, there are some images where the pins are placed relatively high in the metaphysis, crossed at the fracture site, or the fracture is not completely reduced despite open treatment. These may be associated with many causes such as learning curve, the surgeon’s experience, and surgical conditions.

## Conclusion

The results of this study suggest that clinical results of surgical treatment of Gartland type 3 extension fractures were satisfactory. Also no difference between the results of different surgical approaches was found clinically. However, the delay in surgical treatment may cause a number of complications. The choice of surgical approach should be based on the characteristics of fracture and the experience of the surgeon in surgical treatment of displaced supracondylar fractures in children.
